# Transition from paediatric to adult care of adolescent patients with congenital heart disease: a pathway to optimal care

**DOI:** 10.1007/s12471-016-0900-0

**Published:** 2016-09-27

**Authors:** A. M. M. Strijbosch, R. Zwart, N. A. Blom, B. J. Bouma, M. Groenink, S. M. Boekholdt, R. de Winter, B. J. M. Mulder, A. P. Backx

**Affiliations:** 1 Department of Cardiology, Academic Medical Center, University of Amsterdam, Meibergdreef 9, 1105 AZ Amsterdam, The Netherlands; 2 Department of Cardiology, Nursing Staff, Academic Medical Center, University of Amsterdam, Amsterdam, The Netherlands; 3 Emma Children’s Hospital, Department of Paediatric Cardiology, Academic Medical Center, University of Amsterdam, Amsterdam, The Netherlands

**Keywords:** Congenital cardiology, Transition

## Abstract

**Introduction:**

Adolescents with congenital heart disease transition from a paediatric to an adult setting. This is associated with loss-to-follow-up and suboptimal care. Increasing numbers of patients justify a special program. In this study we evaluated the cooperative program between paediatric and adult cardiology departments in a tertiary referral centre.

**Methods:**

In this retrospective study, patients with congenital heart disease with at least one appointment scheduled at the transition program between January 2010 and January 2015 were included. They were seen by a paediatric cardiologist at the age of 15 years in the paediatric department and from age 18 to 25 in the adult department. Demographic and medical data were collected from the electronic patient files.

**Results:**

A total of 193 patients (105 males, 88 females) were identified. Sex distribution was almost equal. Most patients were 18–21 years of age. The largest group, 128 patients (67 %), lived within 50 kilometres of our hospital. Paediatric cardiologists referred 157 (81 %) of patients. General practitioners and cardiologists from outside our centre were important referrers for patients lost to follow-up, together accounting for 9 %. A total of 34 (18 %) patients missed an appointment without notification. Repeat offenders, 16 of 34 patients, formed a significant minority within this group. A total of 114 (59 %) patients were attending school, 46 (24 %) were employed, and 33 (17 %) patients were inactive. Activities are in line with capabilities. A nurse practitioner was involved with the 7 % with complex and psychosocial problems. Moderately severe congenital heart defects formed the largest patient category of 102 (53 %) patients. In 3 % of patients the diagnosis had to be revised or was significantly incomplete. In 30 (16 %) patients, cardiac diagnosis was part of a syndrome. Of the 193 patients, 117 (92 %) were in NYHA class I, with 12 (6 %) and 4 (2 %) patients falling into classes II and III, respectively.

**Conclusions:**

A viable transition program can be built by collaboration between paediatric and adult cardiology departments with the same treating physician taking care of patients between 15 and 25 years of age. General practitioners are important in returning lost-to-follow-up patients to specialised care. Nurse practitioners are essential in the care for patients with complex congenital heart disease.

## Introduction

Due to progress in surgical and medical care, most patients with congenital heart disease (CHD) now survive to adult age. This creates a new and growing group of young patients with unique and specific dedicated needs. For both patients and medical professionals questions arise about limitations, lifestyle, reproduction and what to expect for the rest of their lives. Additionally, upon reaching adulthood, these patients, just like other adolescents with chronic disease, have to transition from paediatric to adult care. If not properly organised, loss-to-follow-up and suboptimal care may result. In the 1990s, the importance of the transition process to the quality of care was already becoming apparent [1]. For this reason, transition programs have been created in medical centres around the world and guidelines have been developed [2]. At the Academic Medical Center (AMC), a tertiary referral centre in Amsterdam, such a program has been in existence since 2004 and was updated in 2010. It is a cooperation between paediatric and adult cardiology. It is physically located in the outpatient department of adult cardiology.

In short, patients 15–25 years of age qualify for the transition program. Older patients are considered to be beyond the transition phase, younger patients are not ready for transition. Patients are able to visit the same physician, a so-called transition-cardiologist, first at age 15 in the paediatric department and then from the ages 18 up to 25 in the adult department. This creates the possibility of patient and treating physician transferring together to the adult setting. Besides medical check-ups, various important topics are discussed during these visits. Some patients are also referred to a specialised nurse practitioner attached to the transition program.

Several years after the initiation of the new program, the time has come for a first evaluation.

## Methods

### Patient selection

Patients with CHD who actually transitioned from paediatric to adult care between January 2010 and January 2015 were selected for this retrospective study. Patients with non-congenital problems such as hereditary or acquired heart disease and with atypical complaints frequently seen in healthy young patients, were excluded. Age at first visit had to be between 15 and 25 years of age. Using these criteria, 193 patients were identified and included.

### Data collection

Demographic and medical data were collected from the electronic patient files.

Data included gender, current age and distance to the AMC in kilometres. Various referral routes were identified: referral by paediatric or adult cardiologists from the AMC or from other hospitals, by general practitioners or by specialists who were not cardiologists from the AMC or from other hospitals. We also looked at the number of times a patient failed to appear at an appointment. School attendance and/or employment was noted. Also, attention was given to the involvement of a specialised nurse practitioner and whether or not evidently more time than usual was spent on the patient. More time than usual was subjectively defined as many hours above usual patient care levels.

Medical data included cardiovascular diagnosis, categorised as a mild, moderate and severe congenital defect (Table 1). In addition, the need to revise the diagnosis, presence of a syndrome and current NYHA class were included. A diagnosis was considered to need revision if the anatomical details differed significantly from those previously reported or were grossly incomplete. NYHA class was determined by combining patient symptoms and results of exercise stress testing.

**Table 1:** Classification of congenital heart defects in adults

**Congenital heart disease of great complexity**
Conduits, valved or nonvalved Cyanotic congenital heart (all forms) Double-outlet ventricle Eisenmenger syndrome Fontan procedure Mitral atresia Single ventricle (also called double inlet or outlet, common or primitive) Pulmonary atresia (all forms) Pulmonary vascular obstructive diseases Transposition of the great arteries Tricuspid atresia Tricuspid atresia Other abnormalities of atrioventricular or ventriculoarterial connection not included above (i. e. crisscross heart, isomerism, heterotaxy syndromes, ventricular inversion)
**Congenital heart disease of moderate complexity**
Aorta-left ventricular fistulae Anomalous pulmonary venous drainage, partial or total Atrioventricular canal defects (partial or complete) Coarctation of the aorta Ebstein’s anomaly Infundibular right ventricular outflow obstruction of significance Ostium primum atrial septal defect Patent ductus arteriosus (not closed) Pulmonary valve regurgitation (moderate to severe) Pulmonic valve stenosis (moderate to severe) Sinus of Valsalva fistula/aneurysm Sinus venosus atrial septal defect Subvalvar or supravalvar aortic stenosis (except HOCM) Tetralogy of Fallot *Ventricular septal defect with* Absent valve or valves Aortic regurgitation Coarctation of the aorta Mitral disease Right ventricular outflow tract obstruction Straddling tricuspid/mitral valve Subaortic stenosis
**Simple congenital heart disease**
*Native disease* Isolated congenital aortic valve disease Isolated congenital mitral valve disease (e. g., except parachute valve, cleft leaflet) Isolated foramen ovale or small atrial septal defect Isolated small ventricular septal defect (no associated lesions) Mild pulmonic stenosis *Repaired conditions* Previously ligated or occluded ductus arteriosus Repaired secundum or sinus venosus atrial septal defect without residua Repaired ventricular septal defect without residua

From: Warnes CA, et al. Task Force 1: the changing profile of congenital heart disease in adult life, 2001. JACC 2001; 37:1170–1175 [8]

## Results

### Demographic data

A total of 193 patients attending the transition program were included. Demographic data are shown in Table 2. As 88 patients (46 %) were female, the sex distribution was almost equal. A total of 156 patients (81 %) were between 18–21 years of age.

**Table 2:** Demographic and general characteristics of included patients

*Total*	*n* = 193	
*Sex*		
Male	105	(54 %)
Female	88	(46 %)
*Age at first visit adult cardiology department*		
Mean	20.12	(SD 1.90)
≤17	2	(1 %)
18–21	156	(81 %)
≥22	35	(18 %)
*Travel distance*		
Mean	42.31	(SD 36.41)
≤20 km	61	(32 %)
21–50 km	67	(35 %)
51–100 km	54	(28 %)
>100 km	11	(6 %)
*Referral routes*		
PCAH	157	(81 %)
PCAE	6	(3 %)
CARH	5	(3 %)
CARE	9	(5 %)
SNCH	5	(3 %)
SNCE	3	(2 %)
GP	8	(4 %)
*No-shows*		
At least once	34	(18 %)
Repeatedly	16	(8 %)
*Daily activities*		
School	114	(59 %)
Work	46	(24 %)
Neither	33	(17 %)

*PCAH* paediatric cardiologists our hospital, *PCAE* paediatric cardiologists elsewhere, *CARH* adult cardiologists our hospital, *CARE* adult cardiologists elsewhere, *NCSH* non-cardiological specialist in our hospital, *NCSE* non-cardiological specialist elsewhere, *GP* general practitioner

Table 2 also shows the distance patients had to travel to the AMC and referral routes. The majority, i. e. 128 patients (67 %), lived within a 50 kilometre radius of the hospital. With regard to referral routes, 157 patients (81 %) were referred to the transition program by paediatric cardiologists from our centre. Cardiologists from other hospitals and general practitioners accounted for 5 and 4 % of the referrals, respectively. The remaining 10 % of patients came from other specialists.

Thirty-four patients (18 %) missed an appointment without notification. This group of 16 repeat offenders were a significant minority.

The distribution of NYHA classification is shown in Table 3. A total of 117 (92 %) patients were in NYHA class I, with 12 (6 %), 4 (2 %) patients and no patients falling into classes II, III and IV, respectively. A total of 114 (59 %) patients were attending school, 46 (24 %) were employed, and the remaining 33 (17 %) patients were neither studying nor working. Consistent with the vast majority of patients being in NYHA class I, most of them worked or attended school. Out of 177 NYHA class I patients only 11 neither attended school nor worked. In NYHA classes II and III this was the case in 4 out of 12 patients and 2 out of 4 patients, respectively (Table 2) The nurse practitioner was involved with 13 patients (7 %) of the transition population.

**Table 3:** Medical characteristics of included patients

*Cardiological diagnosis distribution*		
Severe	43	(22 %)
Moderate	102	(53 %)
Mild	48	(25 %)
*NYHA class distribution*		
I	177	(92 %)
II	12	(6 %)
III	4	(2 %)
IV	0	(0 %)
*Underlying syndromes in patients*		
Down	19	(63 %^a^)
DiGeorge	4	(13 %^a^)
Noonan	2	(7 %^a^)
4p-deletion	2	(7 %^a^)
Turner	1	(3 %^a^)
Williams	1	(3 %^a^)
Fabry	1	(3 %^a^)
Total	30	(16 %^b^)

^a^Of syndromal patients

^b^Of all patients

### Medical data

Table 3 shows the distribution of heart defects. A total of 121 patients (53 %) had congenital defects of moderate severity. Patients with mild and severe congenital heart disease accounted for 25 % (48 patients), and 22 % (43 patients), respectively.

In 6 patients the diagnosis had to be revised or was incomplete because the anatomical details differed significantly from those previously reported or were grossly incomplete. In 30 (16 %) patients the cardiac diagnosis was part of a syndrome, the distribution can be found in Table 3. The most common diagnosis was Down syndrome, occurring in 19 (63 %) of syndromal patients. DiGeorge syndrome accounted for 13 % (4 patients).

## Discussion

The need for a transition program specifically designed for adolescent patients with CHD is widely recognised [3]. In our experience, a transition program can be built by collaboration between paediatric and adult cardiology departments with the same transition cardiologist taking care of patients between 15 and 25 years of age. In most medical centres taking care of patients with CHD, transition programs have been developed. They generally do not differ in their goals. However, they do differ in their structure and size. Ideally, cardiologists responsible for the transition program should be certified in both paediatric cardiology and adult cardiology, as is the case at our hospital. A paediatric cardiologist with experience in adult cardiology is another option seen in transition programs around the world. With additional training in paediatrics, it is also possible for an adult cardiologist to take care of young patients.

At our hospital, patients 15–25 years of age qualify for the transition program, although in the near future this criterion may not be rigidly implemented. As mentioned above, the transition program is physically localised in the Department of Cardiology, an adult setting. A 20-year-old with Down syndrome may be better off in a paediatric setting, whereas a 14-year-old teenager may be more comfortable in an adult environment. One of the main goals of the program is to gradually enable patients to take responsibility for their own health. To achieve this, good information is essential. The focus is always on his or her unique medical history, on the recognition of significant signs and symptoms, and on the importance of regular check-ups. Limitations, issues of lifestyle, employment and reproduction also have to be addressed [4]. Even with the limited amount of time available, it is possible to address these issues during regular visits. As described earlier, patients have the opportunity to visit the transition cardiologist at the department of paediatric cardiology and later again at the department of adult cardiology. In this way, the problem of getting lost between two departments is reduced and patients always know whom to contact if a problem arises.

As expected, 157 (81 %) patients were part of the paediatric cardiology program at the AMC. It is advisable to emphasise the importance of life-long follow-up at an early stage. Four percent of patients (*n* = 8) were referred by their general practitioner. This is an interesting finding. Apparently, general practitioners play a role in preventing patients from getting lost to follow-up after leaving the paediatric environment. Intensifying cooperation with them may be a means to further reduce loss to follow-up.

Most patients lived quite close to the hospital. In case of acute problems, travel time is on average less than one hour. However, being a tertiary referral centre patients come from all over the country. Given the increasing number of adult patients with CHD, cooperation between hospitals in a certain region will become important. In the Netherlands this will be implemented by the CONCARE project: the regional organisation of care for adults with congenital heart disease [5].

It is not unusual for young patients to forget appointments or to give priority to other activities, in large part because they tend to be free of symptoms, even in the face of significant pathology. Besides, many adolescents feel ‘invincible’ and go through a phase of indifference. But it is a missed opportunity for essential check-ups and a missed opportunity for communication. Therefore, the high percentage of no-shows is disturbing and this problem needs to be addressed. At our centre we are now able to send text messages to patients and to remind them of their appointments.

Daily activities are mostly in line with capabilities. Patients without a job or not attending school are an important minority group. Inactivity is associated with NYHA class: the higher the NYHA class, the higher the percentage of patients being inactive at home. Patients with a syndrome and/or significant non-cardiovascular pathology are also part of this group.

It is frequently necessary to invest extra time in young patients; they may have limitations affecting their quality of life, significant residual defects, and uncertain prospects. As congenital cardiology is a rapidly evolving field with increasing therapeutic possibilities, patients previously considered end stage, now ask to revise their medical data. Often, multiple procedures have been performed at different hospitals. This makes an accurate assessment of cardiac anatomy and haemodynamics time-consuming, and sometimes involves second opinions.

Psychosocial issues may be at stake. Social workers are helpful, but they usually lack the medical background, crucial to understanding the problems a patient faces. At our hospital, a specialised nurse practitioner with in-depth knowledge of congenital cardiology is involved in the complex care of these patients.

In a recent survey by the American Heart Association, atrial septal defects and tetralogy of Fallot were found to be the most common congenital heart defects in a grown-up with congenital heart disease (GUCH) population. In our patients, moderately severe pathology, such as tetralogy, was predominant [6]. We encourage adult patients even with mild congenital heart disease to visit our hospital at least once. However, in view of shared care models, close cooperation with regional hospitals is important. In 30 (16 %) of the patients, the cardiac defect is part of a syndrome, a similar prevalence is reported elsewhere [7]. Trisomy 21 and 22q11-deletions are most prevalent in our study.

## Conclusions

A transition program can be built by collaboration between paediatric and adult cardiology departments with the same transition cardiologist taking care of patients between the ages of 15 and 25 years. Important challenges remain, i. e. reduction of lost-to-follow up, reduction of ‘no-shows’, and more systematically addressing important topics such as in-depth knowledge of the medical situation, lifestyle, reproduction, employment [8]. For the patients involved in the transition program we are currently organising separate sessions with a specialised nurse practitioner to address these issues [9]. A transition program may offer a new and challenging pathway for the optimal care of patients with congenital heart disease.
